# PM_2.5_ exceedances and source appointment as inputs for an early warning system

**DOI:** 10.1007/s10653-021-01189-2

**Published:** 2022-02-22

**Authors:** Gladys Rincon, Giobertti Morantes Quintana, Ahilymar Gonzalez, Yudeisy Buitrago, Jean Carlos Gonzalez, Constanza Molina, Benjamin Jones

**Affiliations:** 1 Escuela Superior Politécnica del Litoral, ESPOL, Facultad de Ingeniería Marítima y Ciencias del Mar (FIMCM), Guayaquil, Ecuador; 2 Pacific International Center for Disaster Risk Reduction, ESPOL, Guayaquil, Ecuador; 3 Department of Architecture and Built Environment, University of Nottingham, Nottingham, NG7 2RD UK; 4 Departamento de Procesos y Sistemas, Laboratorio de Residuales de Petróleo, Universidad Simón Bolívar, Caracas, Venezuela; 5 Escuela de Construcción Civil, Pontificia Universidad Católica de Chile, Santiago de Chile, Chile

**Keywords:** Particulate matter, Logistic model, SEM–EDS, EWS

## Abstract

Between June 2018 and April 2019, a sampling campaign was carried out to collect PM_2.5_, monitoring meteorological parameters and anthropogenic events in the Sartenejas Valley, Venezuela. We develop a logistic model for PM_2.5_ exceedances (≥ 12.5 µg m^−3^). Source appointment was done using elemental composition and morphology of PM by scanning electron microscopy coupled with energy dispersive spectroscopy (SEM–EDS). A proposal of an early warning system (EWS) for PM pollution episodes is presented. The logistic model has a holistic success rate of 94%, with forest fires and motor vehicle flows as significant variables. Source appointment analysis by occurrence of events showed that samples with higher concentrations of PM had carbon-rich particles and traces of K associated with biomass burning, as well as aluminosilicates and metallic elements associated with resuspension of soil dust by motor-vehicles. Quantitative source appointment analysis showed that soil dust, garbage burning/marine aerosols and wildfires are three majority sources of PM. An EWS for PM pollution episodes around the Sartenejas Valley is proposed considering the variables and elements mentioned.

**Supplementary Information:**

The online version contains supplementary material available at 10.1007/s10653-021-01189-2.

## Introduction

Particulate matter (PM) is a heterogeneous mix of solid and liquid particles, including chemical and biological fractions, varying in time and space. They are normally composed of different chemical components (elements and functional groups) with different shapes and sizes, but always microscopic. They have different physical properties, such as aerodynamic diameter, surface area, and morphology, characterized by their origin or emission source (Kelly & Fussell, 2012). PM_2.5_ (with an aerodynamic diameter ≤ 2.5 microns) are the smallest particle size that has guideline values in international environmental standards, including guidelines for 24 h exposure time windows (PM_2.5_-24 h) (*e.g.* PM_2.5_-24h_WHO-2021_ = 15 μg m^−3^; PM_2.5_-24h_USEPA_ = 35 μg m^−3^^1^).

Particle properties, including size, can have a major influence on health, being the smallest particle sizes (including PM_2.5_) the ones reporting the most negative impacts on the cardiovascular and respiratory system (Cohen et al., 2017; HEI, 2019). PM could be toxic, carcinogenic, mutagenic, and teratogenic (Apte et al., 2015; Atkinson et al., 2014; Franklin et al., 2008). To characterize the complex mixture of elements within the PM and for source appointment purposes, scanning electron microscopy coupled with energy dispersive spectroscopy (SEM–EDX) is often used (Aragón-Piña, 2011; Huang et al., 2020; Kicińska & Bożęcki, 2018; Miler, 2020; Puławska et al., 2021; USEPA, 2002). Models that can predict the behaviour of PM concentrations, considering sources, such as forest fires, traffic build-up, industrial emission, or multiple weather scenarios, are important inputs when designing early warning systems.

Worldwide, numerous toxicological and epidemiological studies have associated both long term exposure to PM_2.5_ with mortality and morbidity health outcomes: Chen and Hoek (2020) reported that a 10 µg m^−3^ increase in PM_2.5_ concentration is associated with an 8% increase in total mortality (pooled RR 1.08; 95% CI: 1.06–1.09; 25 studies). If the well-being of individuals is addressed by considering both mortality and morbidity, the disability-adjusted life years (DALYs) serve as a measure to quantify both aspects (Murray, 1994). Exposure to ambient PM_2.5_ in 2017, contributed to a burden of 83.10^6^ disability-adjusted life years (DALYs) globally (HEI, 2019). DALYs can be used to estimate the health impacts to exposures to PM_2.5_ for all magnitudes of pollution, independently of threshold or guideline values.

An Early Warning Systems (EWS) is a set of procedures and instruments through which a predictable threat (natural or anthropic) is monitored, and data and information are collected and processed, which will allow future actions to be taken against possible threats (Neild et al., 2007). An PM_2.5_ based-EWS can range from lists of actions to considering simulation models of preventive nature (see Yan et al., 2021; Han et al., 2019; Troncoso et al., 2012; Siata, 2018; Sakellariou et al., 2017; Xu et al., 2017). In all cases, pollution levels in cities force governments to design EWS according to the local context which are usually entangled with established monitoring networks.

Logistic regression is used in air quality analysis to detect when a pollutant concentration is greater than a pre-defined threshold (Kim et al., 2020; Ordóñez et al., 2019), it is also applied to study the relationships between health outcomes and airborne pollutant concentrations (e.g. Bergstra et al., 2018; Ng et al., 2017; Seifi et al., 2019; Ware et al., 2016) as well as to find statistical relationships among PM and environmental/anthropogenic variables (Botero Ortiz et al., 2011; Perez & Reyes, 2002; Upadhyay et al., 2017; Vélez-Pereira et al., 2019; Zickus et al., 2002). Logistic regression provides similar performance to other more complex techniques, such as neural networks or regression splines (Corani, 2005; Dorling et al., 2003; Zickus et al., 2002). Therefore, logistic regression is an acceptable alternative to use to obtain adequate results with an easier interpretation model.

The area of influence for the present study comprehends the Sartenejas Valley, in Caracas, Venezuela, where some studies have been performed to assess PM concentrations, elemental composition and its sources (Guajardo et al., 2010; Morantes et al., 2019, 2021). These studies identified that concentrations and elemental composition of PM were influenced by pollution episodes (namely forest fire events), vehicular traffic activities and the surrounding natural areas. While some research has been carried out on PM at the Sartenejas Valley, the parameters that generate excess PM_2.5_ concentrations are still unknown.

This study proposes a logistic model for exceedances of PM_2.5_ and the source appointment of PM as inputs in a proposal of an Early Warning System of ambient PM_2.5_

## Methods

### Study area

**Fig. 1:**
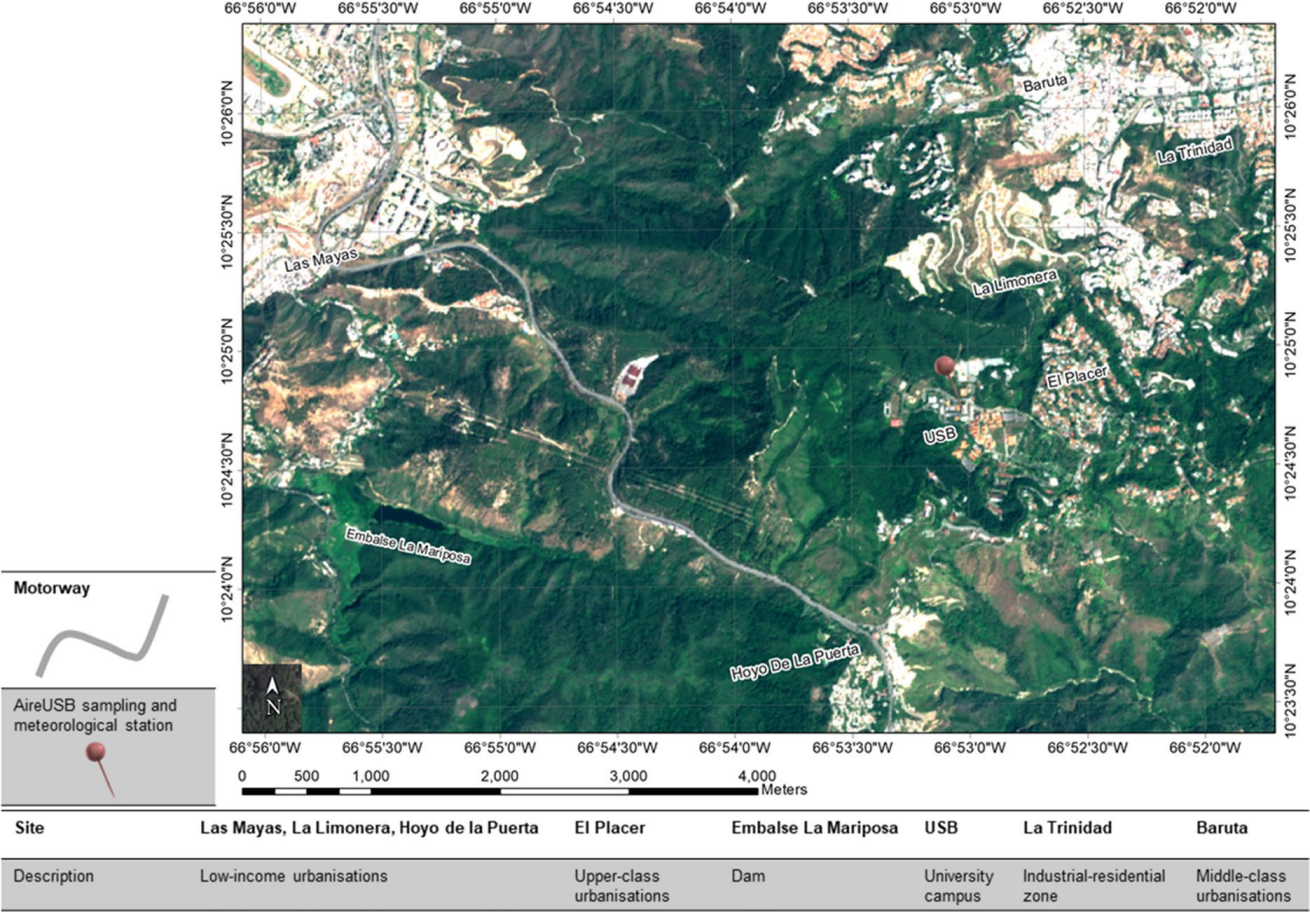
Plan of the study area indicating the location of the PM sampling station

The study area is in the Baruta municipality, southeast of the Caracas Metropolitan Region (CMR). It had 531,627 inhabitants, according to the 2016 census (INE, 2021). The characteristic relief of the Baruta municipality is mountainous mainly because it is located on the limits of the Cordillera de La Costa mountain range (altitudes between 420–1400 m above sea level). The predominant vegetation is dry forest and humid forest, with some mountainous areas reforested with coniferous species, such as on the campus of the Simón Bolívar University (USB). Within the area of influence, it can be found: i) the town of Baruta, that has a mixed use of land (commercial-residential) where artisanal and commercial activities coexist with lower-middle class and lower-class settlements. ii) La Limonera, iii) Hoyo La Puerta and iv) Las Mayas are lower class settlements with a high population density that is constantly increasing. These settlements have shortcomings in essential services, such as solid waste collection. v) La Trinidad is a sector with mixed land use (industrial-residential): the residential use has upper-middle-class and middle-class settlements, and the industrial zone (settled for prox. 50 years) has been displaced by commercial and service activity, but there are still medium and small pharmaceutical and food industries. vi) El Placer, an upper and upper-middle-class settlement. vii) La Mariposa Reservoir supplied the city's water and came into operation in the middle of the last century. iix) The university campus located in Sartenejas Valley, which operates from Monday to Friday, with an enrolment of between 9,500–10,000 students (undergraduate and postgraduate) and a teaching, administrative and service staff of between 3,000–3500 people. Figure 1 shows the location of the sectors mentioned.

There is also a motorway located 2 km west from the Sartenejas Valley, used as the primary vehicular connection between the capital and the west of the country, and an industrial-residential zone 3.5 km northeast. Socioeconomically, the area study shows urban characteristics typical of CMR, where middle-class and upper-class urbanisations border low-income urbanisations. It is highlighted that Ramírez (2012) points out that Venezuela has been showing a trend towards a progressive increase in the lower social strata, where public services are scarce, affecting the operation of the waste management system, causing the inappropriate burning of garbage which emits gases that can be toxic and represent serious fire hazards (Ramos et al., 2012).

The collection campaign of PM_2.5_ was carried out in 2018–2019 in the university campus at an altitude between 1200–1400 m (see Fig. 1). The university campus occupies about 95 hectares, of which 45 hectares are plantations with a high density of pine forest, native forest, secondary shrubland and savanna with diverse shrubs. The geology of the soil occupied by the CMR is made up of metamorphic rocks which were originally sediments, plus some metamorphosed igneous rocks such as serpentinites and amphibolites. Specifically, soils in Baruta are composed of microcline-quartz-muscovite gneiss. Soils in the Sartenejas Valley are typically dominated by fine aggregate of quartz, muscovite, calcite, plagioclase, apatite, and pyrite with porphyroblasts of microcline (Dengo, 1953). Supplemental Figure S1 shows a Geological Map of the CMR, it shows Baruta’s formations as schists and muscovitic, quartz and granatiferous conglomerates, microclinical gneiss and marble limestone (Dengo, 1951).

### Sampling of particles and meteorology

The PM sampling station includes a low volume PM gravimetric sampler and a weather station, located at 9 m altitude to ensure that the air represents the sector (UTM coordinates: 10.412352; − 66.883558 m). The sampling campaign was carried out in three periods: June 30-July 23, 2018; September 17-December 7, 2018; and January 21-April 26, 2019; therefore, sampling includes the rainy season (May–November) and dry season (December–April). In addition, samples were collected for two sampling times: on weekends for 48 h (Sat at 00:00:00 to Sun at 24:00:00) and weekdays for 100 h (Mon at 10:00:00 to Fri at 14:00:00). Sampling times were chosen considering that previous studies at the same sampling station, showed that the mass collected for periods of 24 h could be below the minimum detection limit of the available balance (Morantes et al., 2019). The sampling times were also chosen in order to explore if there is a difference in the trends of concentration of PM_2.5_ taken weekdays (in full work activity) and weekends. Moreover, operational restrictions were considered when defining these sampling times to prevent lack of access to the PM sampling station during restricted hours. The proposed sampling periods and the defined sampling times allow a maximum of 55 PM_2.5_ samples to be taken. All samples were normalized to 24-h concentrations taking the collection time and air volume.

PM_2.5_ sampling was carried out using a Partisol 2000i-D Dichotomous Air Samplers (THERMO Fischer Scientific, MA, US). This sampler operates by splitting PM sample streams into fine and coarse fractions using a virtual impactor. Samplers are typically characterized by their cutpoint, which defines the particle size for which the penetration is 50% (cut point D_50_). The partisol operates at a flow rate of 15.0 L min^−1^ (D_50_ = 2.5 µm) to provide a D_50_ particle size cutoff at 2.5 μm (Loo & Cork, 1988; Thermo Fisher Scientific, 2011). Samples were collected on PTFE (polytetrafluoroethylene) membrane filters of 2 µm pore size, also known as Teflon-CF_2_, 46.2 mm in diameter with a support ring. Meteorological data were collected every 5 min using Davis Instruments 2010 equipment at the AireUSB station, which operated continuously during the sampling campaign.

For gravimetric analysis, a clean filter is weighted and placed in a desiccator for at least 24 h (to control for water absorption) after which it is weighed again. The now dried filter is placed in the Partisol 2000i-D and sampling time is programmed. Once the equipment stops, the sample is removed and stabilized for at least 8 h. After this time, the sample is weighed again, to be immediately placed in the desiccator, for at least 24 h. The dried sample is reweighed. PM mass is obtained by subtraction of dry weights. The samples were placed in aluminium foil, sealed with a plastic cover, and stored/archived in a frost-free refrigerator (< 4C) for future analysis. The filters were weighed on an electronic balance (0.00001-g precision, Ohaus Pioneer PX Analytical Balance). For control of interferences, the temperature of the room is logged for each sample. For control of humidity, humid and dry filter weights were measured in each sample. Widziewicz-Rzońca and Tytła (2020) estimated that PTFE filters have low susceptibility to variations of temperature and humidity due to their hydrophobicity and therefore have the best performance limiting water absorption. The calibration and optimization of the Partisol Dichotomous 2000i-D sampler (Thermo Fisher Scientific) is performed at the start of each year, following the operating manual based on US EPA procedures for measuring particulate matter with low volume equipment. Flow values were checked (and corrected if necessary) at the start of the three sampling periods.

Furthermore, data about relevant emission sources were recorded manually: vehicular traffic and forest fires. Vehicle flow in rush hours was accounted for (07h00- 08h00, 12h00- 13h00, 16h00- 17h00; Monday through Sunday, every-other-day). Forest fires around the Valley were obtained from the university firefighters archive and social media reports (GBUSB, 2019). Moreover, episodes of rain were also recorded because they can generate a cleaning effect by wet deposition (Guo et al., 2014).

Meteorological records indicated that the mean monthly temperature in the Sartenejas Valley remained between 17.4- 22.0 °C, with minimum temperatures between 16.4- 21.9 °C and maximum temperatures between 17.5- 22.1 °C and the relative humidity varied between 71- 98%. The mean wind speed was 2.4 m s^−1^; the predominant wind direction was Northeast (NE). During the sampling months, the maximum rainfall occurred in October (141.80 mm) and the minimum in February (1.16 mm).

### Statistical approach

#### Selection of variables

To discretize the values of PM_2.5_ in binary values, the approach of Pandey et al. (2003) is used, and so the Predicted Variable (PV) is defined as the discretization of exceedances of a threshold of PM_2.5_-24 h. Therefore, defining a threshold that will serve as a cut-off point for the model is imperative. A value of 1 is assigned if the defined threshold is exceeded or 0 if not (a dichotomous format). To discretize the values of PM_2.5_ in binary values the approach of Pandey et al. (2003) is used, and so the Predicted Variable (PV) is defined as the discretization of exceedances of a threshold of PM_2.5_-24 h. Therefore, defining a threshold that will serve as a cut-off point for the model is imperative. In this study, the threshold will be defined later on, based on the magnitude of the concentrations sampled PM, which includes the concentration of PM_2.5_ for the rain and drought season; furthermore, the international guidelines or thresholds proposed will be used as starting points, introducing the DALY concept as relevant within the EWS proposal. A value of 1 is assigned if the defined threshold is exceeded or 0 if not (a dichotomous format).

Previous studies have shown the association between air pollution and two categories of data: meteorological and events. PM concentration can be related to meteorological variables, such as temperature, precipitation, relative humidity, dew point, and wind speed and direction (Morantes et al., 2019; Ul-Saufie et al., 2012). Additionally, it can be related to local air pollution episodes, such as the occurrence of forest fires (also referenced as wildfires), episodes of rain, and vehicular traffic (Chelani, 2019; Liu et al., 2009; Ramos-Herrera et al., 2010; Taheri Shahraiyni & Sodoudi, 2016; Wang & Ogawa, 2015). Thus, a careful selection of the Independent Variable (IV) candidates to be included in the model was carried out based on a knowledge of the physical and chemical behavior of airborne PM. Eleven independent variables were selected for the multivariate analysis and treated as equally important; six meteorological and five related to air pollution events (see Table 1).

**Table 1:** Selected independent variables for the study

Variable (code)	Operational definition
Meteorological	
Temperature^a^ (Temp°C)	Average temperature, AireUSB station for each sampling period [in °C]
Relative humidity^a^ (%HR)	Average relative humidity, AireUSB station for each sampling period [in %]
Dew point^a^ (DW)	Average dew point at atmospheric pressure, AireUSB station for each sampling period [in °C]
Wind speed^a^ (WindSp)	Average wind speed, AireUSB station during each sampling period [in m s^−1^]
Precipitation^a^ (Precip.mm)	Cumulative amount of rainfall, AireUSB during each sampling period [in mm]
Wind direction^b^ (WindDir)	Average wind direction, AireUSB station during each sampling period
Events	
Forest Fire (Forest.F)^b^	Forest fire events reported by the university forest fire department: "1" if sampling event coincides with fire
Rain event^b^ (Rain_y/n)	Rain occurrence, Aire USB station. For rain_time > 50% of sampling time, variable = 1
Vehicle flow^a^ (Veh.T)	Average number of motor vehicles, per hour, that circulate in the study area during the sampling time
Weekday^b^ (wk)	Mon-Fry = 1. Sat-Sun = 0
Holyday^b^ (Holyd)	If holyday = 1

^a^Type: Continuous variable

^b^Type: Non-continuous variable

To establish the individual relationships among the variables and the strength of association, a bivariate analysis is performed using the Student t-test (for parametric variables of different types) and the Pearson’s *r* correlation (as a measure of effect size for parametric variables of the same type). The interpretation of the magnitude of Pearson's correlations follows the guidelines proposed by Ratner (2011). The bivariate analysis between the PV and the IVs is used to assess which variables are retained to include in the logistic model. Significance levels for all the variables were set at *p* < 0.05 (Taylor, 1990). In the logistic model, the nominal levels of significance for the Wald test was *p* < 0.1. To identify possible significant differences between the mean of the PM2.5 concentration obtained for the two sampling times (weekdays and weekend), the t-Student test for independent samples is applied (*α* ≤ 0.05). All analyses were carried out using R statistical software RStudio version 1.1.456.

#### Logistic model

Logistic regression (RLog) is a process of modelling probability used in multivariate analysis. The RLog is a nonlinear technique that uses maximum likelihood estimation to fit the final model. Logistic regression models allow establishing the relationship between a dichotomous qualitative dependent variable with a set of independent explanatory variables, which can be qualitative or quantitative; therefore, RLog models do not provide an estimate of a pollutant's concentration, but rather the likelihood that it will exceed a predetermined threshold. The initial equation of the model is of the exponential type, and its logarithmic transformation (logit) allows its interpretation as a linear function (Eq. 1). The objective of the RLog is to obtain the model that best fits the PV to a set of VIs (Hosmer & Lemeshow, 2000).

1 $$p_{i} = \frac{1}{{1 + e^{{ - (\beta_{0} - \beta_{1} )x_{i} }} }}$$

where *p*_*i*_ probability of *y* = 1 in the presence of covariates *x*; *x*_*i*_ set of *n* covariates; *β*_o_ constant of the model or independent term; *β*_*i*_ coefficients of the covariates.

The RLog, as a type of multivariate regression, must meet certain assumptions that ensure its quality. i) the PV must be dichotomous. ii) the IVs should not have multicollinearity; this is, correlations of *r* > 0.90. iii) outliers should be excluded as they can influence the results of the analysis and lead to incorrect inferences; the outliers are identified using the interquartile range (IQR) criterion. Moreover, a RLog does not require a linear relationship between the dependent and independent variables. Homoscedasticity is not required. The error terms (residuals) do not need to be normally distributed; however, when there is a normal distribution in the predictors, the solution may be more stable (Hosmer & Lemeshow, 2000).

Statistical validation or adjustment of the logistic regression model is obtained using the chi-square likelihood statistic with a significance level of *p* < 0.05. To obtain this, the prediction values are compared with the values observed in two moments for the model: 1) without variables and 2) with the predictor variables. The likelihood value must decrease significantly, and when the model predicts adequately, it will tend to zero (using a cutoff point of *p* < 0.05) (McSharry et al., 2009).

The logistic model is evaluated using summary statistics in a contingency-classification table; this table summarizes the model’s goodness (McSharry et al., 2009). The classification table indicates the *absolute frequency* (total number of observations), the *correct classification* percentages—observed and predicted cases of exceeding and not exceeding the threshold- and the *holistic success rate*. The contingency-classification table can also be used to measure the sensitivity and specificity of the model. The model is then validated using classification errors (false positives and false negatives). The holistic success rate is calculated based on the table’s main diagonal (correct classifications).

### Source appointment

#### SEM–EDX analysis

The scanning electron microscopy (SEM) analysis was carried out with a computer-controlled scanning electron microscope (JEOL model JSM6390) coupled with an energy dispersive spectroscopy (EDX) (Inca software). The SEM working conditions were set at an accelerating voltage of 30 kV. The images were recorded at two different magnifications, 1000 × and 500 ×.

Sections of ~ 2 cm^2^ of filters were cut and coated with a thin layer of gold (Au) for SEM–EDX samples. Microscope magnifications of 100, 50, 20, and 10 µm were used. About fifteen random particles were selected on each field filter, giving approximately fifteen manually characterised particles per filter. The EDX spectra of blank filters were also measured and subtracted from the samples. The blank filter’s elemental composition showed an approximate 1C:2F ratio and coated in gold. The contributions of C, F and Au, were manually subtracted during the EDX spectra evaluation. The weight percent of each element present in the spectrum was identified and normalised to 100%wt.

#### PM physicochemical characterisation

Both elemental composition and particle size are properties that can be related to an aerosol’s origin; however, elemental composition is a more robust and reliable indicator for source appointment. A qualitative and quantitative analysis of elements was used to propose plausible particle groups taking into account (i) the elements found and their %wt; (ii) morphology and; (iii) bibliographic review, particles groups reported for the Sartenejas Valley (Morantes et al., 2021; Rincon et al., 2019).

The D_50_ cutoff point for 2.5 µm ensures a collection of 50% of aerosols with aerodynamic diameters of less than 2.5 µm, allowing 50% of the largest particles to reach the filter. There is the possibility of encountering particles larger and smaller than 2.5 µm in the stream. In this investigation, the SEM–EDS analysis included the possibility of analyzing any of the particles present in the Teflon filter. The particles’ selection criteria for the SEM–EDS analyses were: non-agglomeration of particles for easy observation, morphology, composition, and size. Furthermore, samples with PM concentrations above and below the threshold were analyzed separately using SEM–EDS.

#### PM quantitative source appointment

Principal component analysis (PCA) and hierarchical cluster analysis (HCA) have been widely used in the study of particulate matter for identifying the relationships between particles and their sources (Błaszczak, 2018; Genga et al., 2012; Tsitouridou et al., 2013). The elemental composition of particles (%wt) obtained with SEM–EDX was used for source appointment applying PCA and HCA. Factor loading was set for higher than 0.7 absolute. Varimax rotation was used as the rotation method for PCA through the Spearman correlation. Likewise, HCA was used for comparing the results obtained from the PCA. XlSTAT statistical software was used.

## Results and discussion

### Logistic model

#### PM_2.5_ concentrations

Forty-one (41) samples were collected in 21 weeks. Table 2 presents the summary statistics of PM_2.5_-24 h concentrations for the two collection sampling times: the 48-h and the 100-h, which were normalized to 24 h (see “Sampling of particles and meteorology” section). The t-Student test applied to the 48 h and 100 h data series did not show a significant difference in the means of the concentrations taken at each collection time (See footnote Table 2). Furthermore, FTIR analysis of samples collected for these two sampling times at the same station during the rainy season in 2018, showed that there were no differences between the magnitude of signals and functional groups detected in samples collected during weekdays and weekends (Morantes et al., 2021). The above shows that the samples collected in the two sampling times can be directly compared.

**Table 2:** PM_2.5_-24 h Concentrations sampling-campaign 30 Jun–23 Jul 2018; 17 Sep–07 Dec 2018; 21 Jan–26 Apr 2019- Sartenejas Valley (UTM coordinates, 10.412352; − 66.883558 m)

Collection period (h)	PM_2.5_-24 h (µg m^−3^) (95% CI)	Standard deviation (μg/m^3^)	PM_2.5_-24 h máx. (μg/m^3^)	PM_2.5_-24 h mín. (μg/m^3^)	No. samples
48	9.30* (2.31–22.16)	6.67	23.15	2.22	17
100	9.97* (2.83–34.11)	9.58	50.00	2.31	24
			Total		41

^*^ Means are not statistically different (t-student test for difference in means *p* < 0,86)

**Fig. 2:**
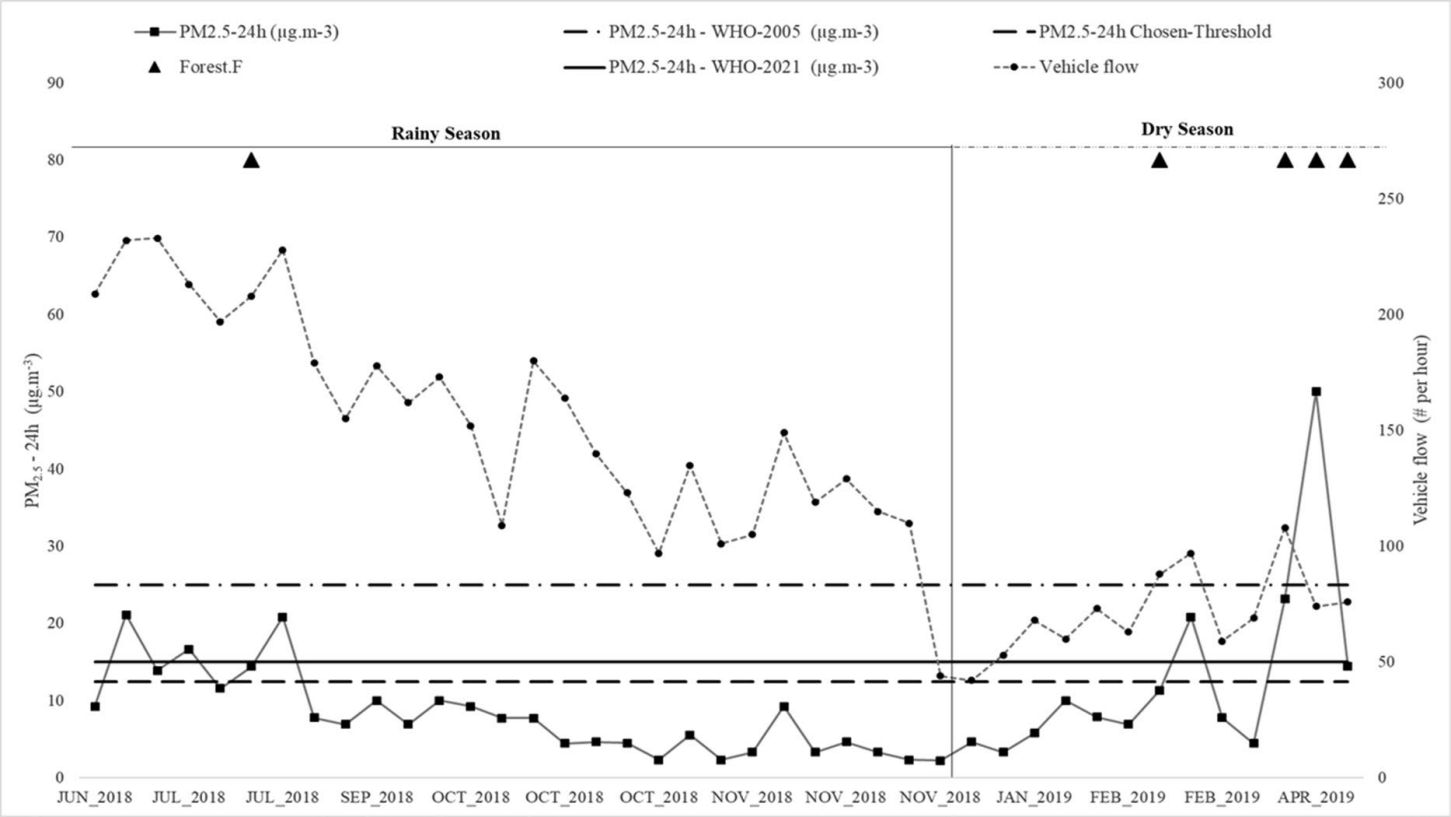
Sartenejas Valley PM_2.5_-24 h concentrations and events (June 2018 to April 2019)

Figure 2 shows the concentrations of PM_2.5_-24 h for the sampling-campaign, along with the vehicle flow, forest fire events, and the rainy and dry seasons. This graph shows that the concentration of PM_2.5_-24 h increases with vehicular traffic (a *moderate* effect size of *r* = 0.37; *p* < 0.05) and forest fires events (a *moderate* effect size of *r* = 0.44; *p* < 0.05). On the other hand, PM_2.5_-24 h correlates negatively with the rainy season (a *moderate* effect size of *r* =  − 0.56; *p* < 0.05). Comparing the results to those of other studies confirms the relationships between PM_2.5_ concentration and the physical events described in “Selection of variables” section (Phuleria et al., 2005; Jaffe et al., 2008; Yoo et al., 2014; Blanco-Becerra et al., 2015; Sahanavin et al., 2018; Morantes et al., 2019).

In both sampling times, average PM_2.5_-24 h concentration levels are below the WHO’s 24 h guideline value of 25 μg m^−3^ (2005) and 15 μg m^−3^ (2021); however, maximum values do exceed the guidelines, mostly in dates associated with multi-day wildfires (08-12Apr 2019) (See Fig. 2): there is a 1% exceedance to WHO-2005 guideline, 15% exceedance to WHO-2021 guideline, categorizing the Sartenejas Valley with low levels of PM_2.5_. PM_2.5_-24 h concentrations are like those reported in 2015 for this sector (Morantes et al., 2019).

To further explain the influence of wildfires on the concentrations of PM_2.5_, sampling values for PM_2.5_-24 h taken at the same sampling station, for the year 2014–2015 are presented as supplementary data (Figure S2). During that sampling-campaign, PM_2.5_-24 h exceeded the WHO-2005 and WHO-2021 guideline values during multi-day wildfires (April–May 2015).

Since a safe level of PM_2.5_ concentration has not been established yet, mortality and morbidity health effects could still be related to low exposures to this pollutant. A study of the association between long-term exposure to low-level PM_2.5_ (annual average PM_2.5_ concentrations from 1.6- 9.0 μg m^−3^) and mortality, showed that 1 μg m^−3^ increase in annual PM_2.5_ could be associated with 2.02% (95% CI 1.41–2.63%; *p* < 0.01) increases in total mortality in Queensland (the second largest state in Australia) and a 5.65% (95% CI 4.08–7.25%; *p* < 0.01) increase in total mortality quantified particularly for Brisbane (largest city in the state) (Yu et al., 2020).

To further study the impact of the magnitude of the concentrations of PM_2.5_-24 h of this study, the values are used to compute DALYs in order to quantify health burden independently of any threshold or guideline value, following the method of Fantke et al. (2019). The results in Table 3 indicate that average concentration of PM_2.5_-24 h is related to a burden of 6.57 DALYs per 1,000 people (95% C.I. 1.41–7.04). The global burden of disease attributable to ambient air pollution (PM_2.5_) shows an estimate for Venezuela of 8.32 DALYs per 1,000 people (95% U.I. 6.97–9.77) (Cohen et al., 2017). When accounting for the 95% C.I. of sampled PM_2.5_ concentrations, it can be noticed that the upper limit shows burdens higher than the equivalent burden for the recommended PM_2.5_ global guidelines. Concentrations of PM_2.5_ measured in this study might already show a significant burden of disease for the country, regardless of whether a threshold is exceeded.

**Table 3:** Burden of health, based on DALYs, for PM_2.5_-24 h Concentrations

	PM_2.5_-24 h (µg.m^−3^)^±^	DALYs*°^±^
Average	9.35 (2.57–28.14)	6.57 (1.41–7.04)
Min	2.22	NA
Max	50	7.12
Chosen threshold	12.5	6.76
WHO-2005 guideline	25	7.01 (6.14–7.91)
WHO-2021 guideline	15	6.85 (5.82–8.00)
Cohen et al. (2017) (95% CI)	15–30	8.32 (6.97–9.77)

*Per 1,000 population

^±^(95% CI)

°All-Cause Mortality based-DALYs

NA. Value below the TMREL for PM_2.5_ curve

Once the values of the concentrations of PM_2.5_-24 h during the sampling campaign are known, it is possible to define the RLog threshold for the PV. For statistical purposes, to capture abnormal concentrations and considering the WHO-2005 24 h guideline value (PM_2.5_ < 25 μg.m^−3^) a the cut-off point value of PM_2.5_-24 h = 12.5 μg m^−3^ was decided as the maximum threshold (PM_2.5_exc_12.5_) for the logistic analysis. This value is representative of the mean and median of the concentrations, being 50% of the WHO-2005 recommended guideline value. Previous research had proven that the exceedance limit for statistical purposes could be chosen according to each investigation’s particularities and specific levels of pollution. For Helsinki, Finland, a city with low pollution, the WHO-2005 threshold value (PM_10_-24 h > 50 μg m^−3^) was used for the limit of the exceedance model (Zickus et al., 2002). Conversely, studies in highly polluted locations chose much higher limits in their models, values even higher than those suggested by environmental regulations. For example, for Santiago de Chile, the threshold for modelling PM_10_-24 h exceedances was 240 μg m^−3^ (Pérez & Reyes, 2002; Alvarado et al., 2010); this value is higher than that of its 1992 national regulations (150 μg m^−3^). However, this value responds to National Environmental Commission recommendations, which defined four levels of PM_10_ concentrations to make administrative decisions at the time of severe episodes: good, 0–193 μg m^−3^; alert, 194–239 μg m^−3^; pre-emergency, 240–329 μg m^−3^; and emergency, > 330 μg m^−3^. In Hangzhou, China, for a model of PM_1_-24 h (PM_1_, PM less than or equal to 1 μm; particle size with no environmental atmospheric standards for determining the “high” and “low” levels), the exceedance limit was PM_1_-24 h > 150 μg m^−3^, showing that the threshold to be chosen to model excesses to a specific value of PM can be intuitive, not even based on statistics like the mean or median (Pandey et al., 2003).

When assessing the current WHO-2021 PM_2.5_-24 h guideline, it can be noticed that the maximum threshold for the logistic analysis chosen in this paper is about 2.5 absolute points (in μg m^−3^) below the recommended value, indicating that the magnitude of the cut-off point value chosen before the publication of the new WHO-2021 guidelines, was reasonable. Furthermore, when considering an EWS, the DALY concept gains relevance as it allows for the quantification of health burden for any magnitude of exposure; this is particularly important when working in low-PM_2.5_ areas when guidelines proposed by organisms such as the WHO, or thresholds regulated such like the USEPA, might not be exceeded and therefore, no quantification of health is accounted for.

Figure 2 shows that 9 of 41 samples have exceeded this threshold (12.5 μg m^−3^) for the year of study (2018–2019) (21% exceedances). This tendency can be compared with the sampling values shown in Figure S2 (Supplementary material) for 2014–2015, where the threshold of 12.5 μg m^−3^ is exceeded in the dry season for 17 of 48 samples.

#### Variables

Table 4 shows the results of applying the Student *t*-test between PM_2.5_exc_12.5_ and all continuous IVs for variable retention. Based on their significance, mean air temperature, relative humidity, dew point, wind speed, and precipitation are eliminated, and only vehicle flow is retained (at *p* < 0.05 level; mean = 181.667 veh h^−1^). As vehicular flow increases, there are more significant emissions from exhaust pipes and more resuspended soil dust by friction between tires and pavement (Sahanavin et al., 2018).

**Table 4:** Student *t* test between PM_2.5_exc_12.5_ and continuous independent variables (IVs)

Continuous IVs	Mean PM_2.5exc12.5_ = 1	Mean PM_2.5exc12.5_ = 0	t	*p*
Temp °C	19.865	20.117	− 0.4929	0.6257
%HR	87.178	87.818	− 0.5786	0.5672
DP	17.818	17.912	− 0.1832	0.8559
WindSp	2.474	1.959	1.0143	0.3522
Precip.mm	1.132	1.863	− 1.5903	0.1222
Veh.T	**181.667**	**124.846**	2.5378	0.0166

1: Threshold exceedances; 0: below the threshold

Significant mean differences in bold, *p* < 0.05. See Table 1 for a description of independent variables

Contingency tables between PM_2.5_exc_12.5_ and non-continuous variables (nIVs) are shown in Table 5. These tables allow us to identify the percentages of expected-predicted coincidences between the nIVs and the VP. Low percentages indicate weak relationships (independent values). The day of the week, holiday, and wind direction were discarded because they did not present independent values. Some expected classifications are noted for forest fires − Forest.F (0–0 expected-predicted coincidences = 96%) and rain episodes − Rain_y/n (1–0 expected-predicted coincidences = 76%). The occurrence of forest fires is associated with higher concentrations of PM_2.5_: Jaffe et al. (2008) and Phuleria et al. (2005) demonstrate the marked influence of forest fires in increasing PM_2.5_ concentrations. Likewise, the occurrence of rain is associated with lower PM levels; Yoo et al. (2014) and Blanco-Becerra et al. (2015) discuss the high sensitivity of PM air pollution to wet deposition and find a statistically significant reduction. However, since the relationship between the PV and episodes of rain showed less than 90% expected-predicted coincidence, this nIV was discarded for the RLog. As a result of the analysis of the IVs, the variables selected to be considered by the model are vehicle flow and forest fires.

**Table 5:** Contingency table between PM_2.5_exc_12.5_ and non-continuous independent variables (nIVs)

nIVs		PM_2.5exc12.5_	
		0	1
wk	0	11	3
	1	15	3
Holyd	0	25	5
	1	1	1
Rain_y/n	0	6	4
	1	20	2
Forest.F	0	25	4
	1	1	2
DirW	NNE	5	1
	NE	13	2
	ENE	8	3

#### Model

For the construction of the logistic model, 32 of 41 samples were used as PV (see “Selection of variables” section). We found 9 outliers that were removed. Table 6 shows the result of the logistic regression model. Positive β coefficients (see Eq. 1) for vehicular flow and forest fire are 0.1313 and 16.7980, respectively, indicating an increased probability of exceeding the PM_2.5exc12.5_ threshold. The concentration of PM_2.5_-24 h exceeded the chosen threshold of 12.5 μg m^−3^ when any of the two anthropogenic events (forest fires or high vehicle flow) occurred; see Table 6. In the model, forest fires have a greater influence on the exceedances than high vehicle flow. These relationships are consistent with those shown in the correlation analysis given in Tables 3 and 4. The mathematical expression of the logistic model is now defined as

2 $$p_{i} = \frac{1}{{1 + e^{{ - \left( {27.4957 - \left( {16.7980{\text{Forest}}.F + 0.1313{\text{Veh}}.T} \right)} \right)}} }}$$

*p*_*i*_ probability that PM_2.5_ exceeds the threshold when each independent variable’s values are equal to their mean value.

**Table 6:** LOGIT model

Variables	Β	SD	Wald Test	*p* value
Constant	− 27.4957	15.8145	− 1.739	0.0821
Veh.T	0.1313	0.0744	1.766	0.0775
Forest.F	16.798	9.7462	1.724	0.0848

*Veh.T* vehicle flow. *Forest.F* occurrence of forest fires

*N* = 32; − 2loglikelihood =  − 7.52; Chi-cuadrado (2 g.l.) = 23.3728 (*p* = 0.00)

β standardized beta coefficients; SD, Standard deviation; 9 samples were discarded for a better fit of the model

Table 7 presents the classification table of the logistic model. It shows the observed group (rows) and the predicted group (columns) with a sensitivity of 96% and a specificity of 83%. These values show that the model successfully classifies both positive and negative responses. Validation using the indicators of false positives and false negatives shows 16% of false positives and 4% of false negatives, which indicates < 20% of classifications are wrong. Overall, the model presents a holistic success rate of 94%.

**Table 7:** Classification table of the LOGIT model

Observed [PM_2.5exc12._]	Predicted [PM_2.5exc12_]		Correct classification (%)
	0	1	
0	25	1	96.16
1	1	5	83.33
	Holistic success rate		93.75

The cutoff value was 0.500; False positives 1 of 6: 16.67%; False negatives: 1 of 26: 3.85%

Holistic success rate = [25 + 5] / [32] = 93.75%

### PM physicochemical characterisation and source appointment

#### By event influence

**Fig. 3:**
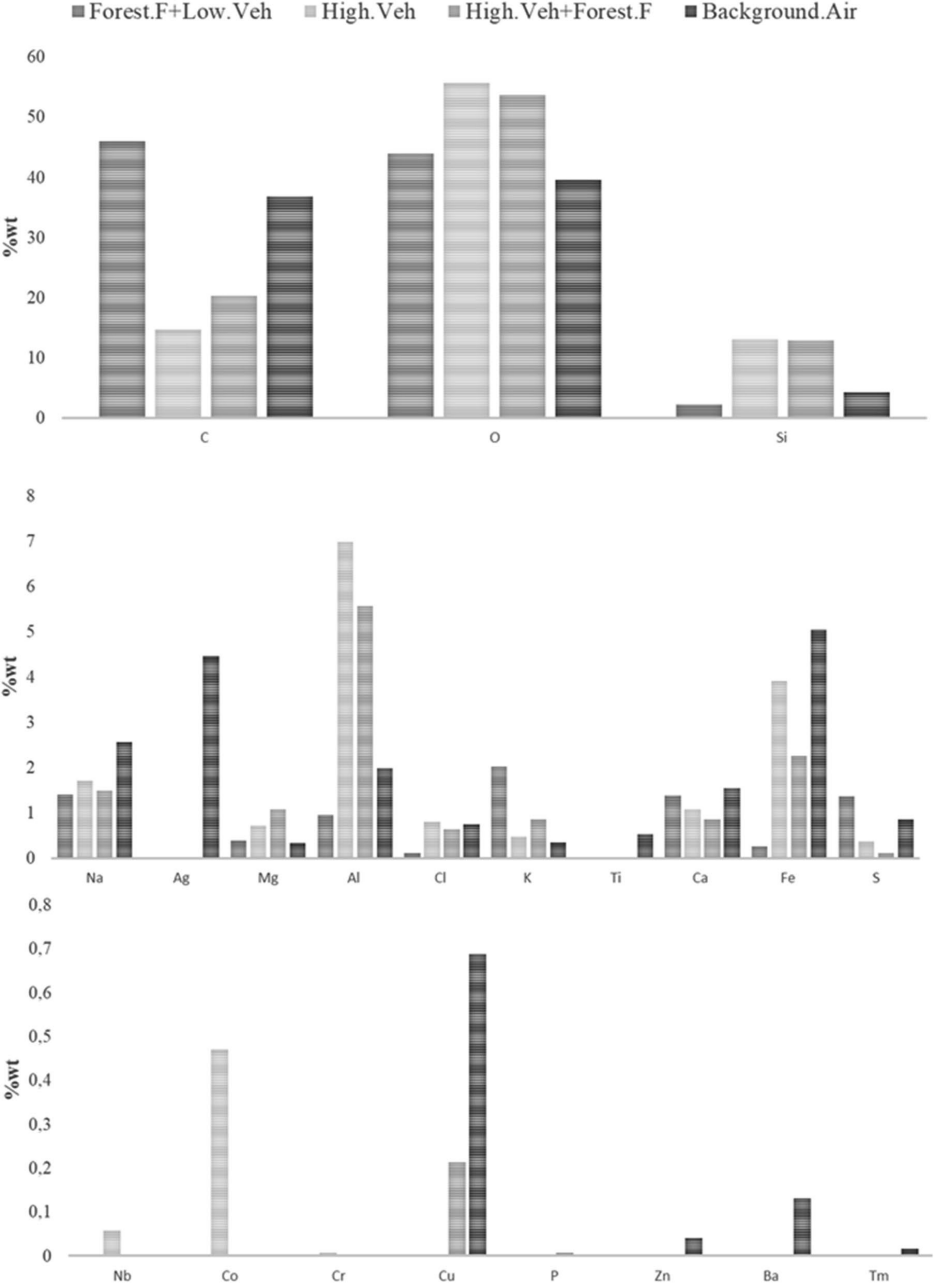
Elemental composition of PM for four categories (Up: predominant elements; Medium: lower proportion elements; Low: traces elements). *Note* [Forest.F + Low.Veh]: Forest fires with low vehicle flow; [High.Ve]: high vehicle flow without forest fires; [High.Veh + Forest.F]: High vehicle flow with forest fires; [Background.Air]: samples with lowest PM concentration, lowest vehicular flow, and without fires

We analysed two hundred airborne particles from twelve samples collected by SEM–EDX. From this analysis, it was established that the particles contain different elements. Therefore, to analyse the PM, they are categorised as geogenic, metallic, C-rich and, secondary aerosols; in turn, the various categories were organised into groups and subgroups/possible compounds (see Fig. 3).

The samples were categorized according to the occurrence of an event into i) forest fires with low vehicle flow (Forest.F + Low.Veh); ii) high vehicle flow without forest fires (High.Veh); iii) high vehicle flow with forest fires and (High.Veh + Forest.F); iv) low vehicular flow without fires (Background.Air)- used as background or reference air. The occurrence of rain-days was analyzed and discarded for not representing a specific category. Samples with the highest PM_2.5_-24 h concentrations were used for categories i, ii, and iii (*N* = 14), while samples with the lowest PM_2.5_-24 h concentration were used for category iv (*N* = 5).

We analyzed 162 micrographs from the first three categories and 53 micrographs from the fourth category using SEM–EDS (see “Source appointment” section). Figure 3 shows the average percentage weight (%wt) of each element for the four categories. It shows that C, O, and Si are the predominant elements; Na, Mg, K, Ca, Fe, S, and Cl are present in lower proportions, and Ti, Cu, Nb, Co, Cr, and P are present in traces. The %wt distribution is similar to other studies made in Argentina (Giuliani et al., 2017) and in the Middle East (Zeb et al., 2018). In this study, Ag was only present in the reference category. It is noteworthy to mention that when applying SEM to samples coated with Au, there is a possibility of interference from the Au coating when identifying the S signal during the EDS analysis. Therefore, the findings of the presence of S must be interpreted with caution.

**Fig. 4:**
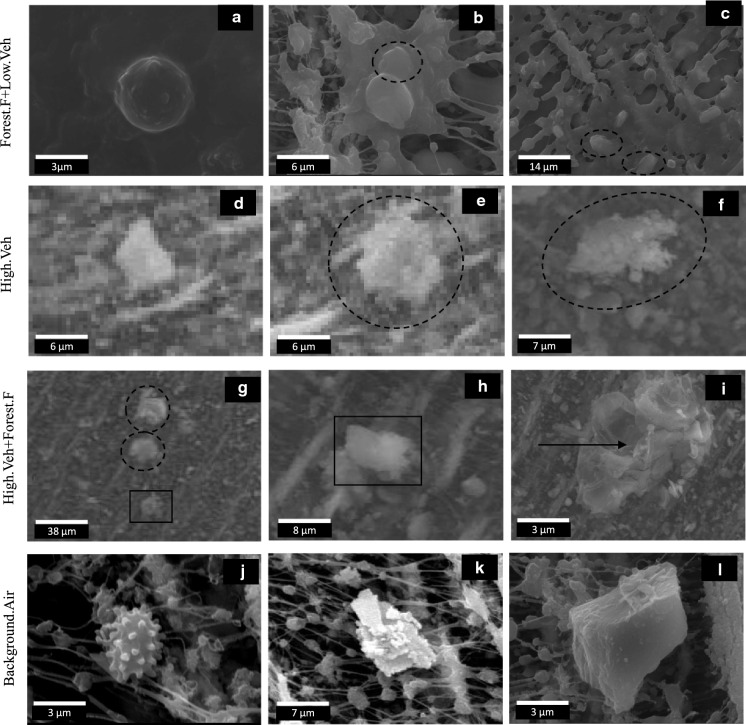
SEM photomicrographs for proposed particle groups: **a** black carbon **b** carbonaceous mixture with S; **c** carbonaceous mixture; **d**, **e** Mineral dusts; **f** Co-containing; (g_dotted-circles) high carbon particles; (**g**, **h**_squares) Carbon-rich quartz; **i** Cu-containing: **j** Biological; **k** Ag particle; **l** Natural quartz. *Note* [Forest.F + Low.Veh]: Forest fires with low vehicle flow; [High.Ve]: high vehicle flow without forest fires; [High.Veh + Forest.F]: High vehicle flow with forest fires; [Background.Air]: samples with lowest PM concentration, lowest vehicular flow and without fires

Category (i) Forest.F + Low.Veh, contains the highest relative percentage of C and K. Both are considered tracers of biomass burning (Amici et al., 2011). This category has the lowest proportion of Si-Al, characteristic of soil dust (Zeb et al., 2018). Category (ii) High.Veh, and category iii) High.Veh + Forest.F, have a high content of Al-Si-Fe–O, associated with soil dust resuspension, probably due to high vehicular traffic (Pipal et al., 2014). Furthermore, category (iii), from days with forest fires, shows more complex PM with C-O–Al-Si mixtures and with a higher carbon content. Category iv) Background.Air presents mixtures with a high C-O contents associated with biogenic aerosols (Zeb et al., 2018), Al-Si-Fe-Ca-O related to mineral dust (Zeb et al., 2018), Na-Cl that might be associated with marine aerosols (Wang et al., 2017) or rubbish/garbage burning (Li et al., 2012), and mixtures of Fe-Ag that are associated with the metal structures of the campus buildings (Aragón-Piña, 2011). Regarding the rubbish/garbage burning in the study area, the municipal garbage collection system does not always reach all sectors of the communities around the Sartenejas Valley, pushing the population to burn their garbage open air as a common practice for final disposing in the city (Ramos et al., 2012). Ag particles might not have been found in the other categories because of their low abundance in the samples relative to other elements and related to the events. Figure 4 shows three SEM–EDS images for each of the four categories studied. The first row shows images from category i, which shows that SEM–EDS of forest fire sample filters tended to be saturated. Figure 4a shows a rounded C-O particle that is carbon-rich. These particles are related to exhaust emissions from motor vehicles and biomass burning (Pachauri et al., 2013; Shi et al., 2003; Zeb et al., 2018). Figure 4b presents a carbonaceous mixture with Pachauri et al. (2013) mentioned that sources of these types of particles include burning of fuels and biomass, as well as waste incineration. The morphology of carbonaceous particles is influenced by the type of fuel, burning conditions and atmospheric processes. Figure 4c has two similar particles containing C-S-K–O; K is a tracer of forest fires (Amici et al., 2011). The S present in the particles is possible because SO_2_ can be adsorbed on mineral particle surfaces (Zeb et al., 2018) or from the adsorption and secondary phases of SO_4_ present in the environment (Boev et al., 2013). SO_2_ is presumed to come from the burning of fossil fuels.

The second row of Fig. 4 presents micrographs from category ii. Figure 4d, e show particles containing Al–Si–O–Fe. These are mineral dust or mixtures of aluminosilicate particles with other crustal elements such as Fe, K, Ca, Na, and Mg, probably from the earth (Pachauri et al., 2013; Zeb et al., 2018). These aerosols are mostly associated with resuspended soil dust from the vehicular motor activity and typical of the study area (Rincon et al., 2019). Figure 4f shows a mineral dust mixture with metallic elements. Road dust resuspended by vehicular activity could be rich in Fe, Cu, Co, and Ni as they could originate from vehicle exhaust and brake wear (Wang et al., 2013; Rincon et al., 2019).

Particles from category iii (third row) have a complex mixture of elements. Figure 4g shows dotted circular aerosols with content of ~ 90%wt of C and O. Its major elements are Al-Si and contain traces of Na-Mg-Cl-K-Ca-Fe-S. These can be grouped as biogenic aerosols or carbonaceous mixtures with S. Carbon-rich quartz in squares in Fig. 4g, h. These particles—with C-O-Si as major elements—are a mixture of carbonaceous and inorganic elements with varying amount of soil-related components, such as Si, Na, Mg, K, Cl, Ca, and Al, forming complex aggregates with variable sizes and morphologies (Pachauri et al., 2013; Weinbruch et al., 2010; Zeb et al., 2018). High carbon aluminosilicates and carbonaceous mixtures with S have been interpreted as dust-soot aggregates: soot (black carbon) have been reported to be (externally) mixed with dust, and adhered to the surface of the clay, aluminosilicates, quartz, or Fe-mixtures (Cvetković et al., 2012; Gao et al., 2007; Quinn et al., 2004). This category presents complex C–O–Al–Si mixtures with high carbon content. The C present in the mixtures could represent the influence of forest fire smokes mixing with the resuspended soil dust. Figure 4i displays a Cu-containing particle; the arrow points to the spot where EDS was taken). Cu is associated with wearing brakes and automobile oil pumps or corrosion of metal parts coming in contact with oil (Sah et al., 2019). These particles are believed to be generally associated with road dust resuspended by motor vehicles.

Category (iv)- the fourth row represents surrounding green areas: biological-natural particles are found in this category (see Fig. 4j). Such biological particles include microorganisms and organic fragments of all varieties of living matter (namely, viruses, bacteria, fungal spores, pollen, plant debris, animal matter, protozoa, fungi, and/or fragments of leaves) (Pachauri et al., 2013; Zeb et al., 2018). Ag-rich particles (Fig. 4k) are associated with steel and metalworking emissions (Aragón-Piña, 2011). In this case, they might be related to welds from workshops present on campus and the metallic material of the buildings’ façade. Figure 4l shows a particle of natural origin containing predominantly silicon, classified as quartz (SiO_2_), probably from the resuspension of soil dust and earth’s crust (Pachauri et al., 2013). When analyzing the SEM–EDS of samples with low concentrations of PM_2.5_, it was found that they mainly come from the green areas surrounding the campus and soil dust and there may be an influence on the local activities of the university campus.

The elemental and morphological analyses established that according to the events observed, PM could have complex mixtures of elements, including high proportions of Al and Si and traces of S. This could be important when determining the relationships between PM exposure and health outcomes. For example, Franklin et al. (2008) found an increase in non-accidental mortality when the PM_2.5_ mass contained a higher proportion of aluminium (IQR = 0.58%), arsenic (0.55%), sulfate (0.51%), silicon (0.41%), and nickel. (0.37%). Similarly, Gehring et al. (2015) computed morbidity health effects (associations presented as odds ratios (OR) and relative risks (RR) for asthma, hay fever and allergic reactions to the components of PM_2.5_ in children. S and K in PM_2.5_ were positively associated with asthma; Zn and K in PM_2.5_ were positively associated with rhinitis. These findings show there is a relationship between the elemental composition of PM and specific mortality and morbidity health effects. The SEM–EDS analysis showed that when the threshold was exceeded, the predominant sources of aerosols were forest fires and the flow of motor vehicles; both events are modelled in the logistic model; see “By event influence” section.

#### Quantitative source appointment

PCA was performed with PM´s elemental composition (% wt) obtained by SEM–EDX analysis of 332 particles of PM_2.5_. Six factors explain 75% of the variability (Table 8). The elements Ag, Nb, Co, Cr, P, Zn, Ba and Tm were discarded because of their low presence in particles.

**Table 8:** Factors of the main components of the composition of particulate material rotated by the Varimax method

Factor	PC1	PC2	PC3	PC4	PC5	PC6
Source Element	Soil	Garbage burning || marine aerosols	WildFires	Secundary particles	Soil dust || Metallic sources	Vehicular activities
C	− **0**.**9385**	0.0098	− 0.0269	0.0260	− 0.0098	0.0081
O	**0**.**8574**	− 0.1001	− 0.0278	0.0133	− 0.1416	− 0.0602
Na	0.0026	0.5799	0.4554	0.1474	− 0.1506	− 0.1313
Mg	0.2824	0.1527	0.5641	− 0.0992	0.1777	0.2341
Al	**0**.**8059**	0.0513	0.1362	− 0.1263	− 0.0460	0.0575
Si	**0**.**8040**	0.0154	0.0838	− 0.0666	0.0755	− 0.1140
Cl	− 0.0039	**0**.**9134**	0.0169	0.0039	0.0645	− 0.0009
K	0.0583	0.0197	**0**.**8582**	0.1157	− 0.0217	0.0035
Ti	− 0.0030	− 0.0168	0.0199	− 0.0405	**0**.**9476**	− 0.0104
Ca	0.0548	0.3671	− 0.0466	0.5815	0.0334	0.4905
Fe	**0**.**7158**	0.1212	0.2235	0.0341	0.3913	0.0511
S	− 0.1437	− 0.0159	0.1078	**0**.**9091**	− 0.0549	− 0.0665
Cu	− 0.0984	− 0.1077	0.1003	− 0.0099	− 0.0140	**0.8852**
Variability (%)	27	11	10	9	9	9
Accumulated (%)	27	38	48	57	66	75

Bold indicates highest loading for each element ( >|0.7|)

The first factor (PC1) explains 27% of the total variance and it shows a high positive correlation for Si, Al, O and Fe, which in term correlated negatively with C. Elements Si, Al and O are characteristic of aluminosilicates and other unreacted mineral dust. When combined with Fe, they are classified as clay, such as almandine or kaolinite common in soils of tropical regions (Kothai et al., 2011; Satsangi & Yadav, 2014). PC1 represents a mixture of re-suspended soil dust with a mostly non-mineral carbon source.

The second factor (PC2) explains 11% of the total variance with Cl as the main element. Cl presence in particles have been associated with sea aerosols, the combination of these aerosols with soil dust, or rubbish/garbage burning (Almeida et al., 2006; Kothai et al., 2011; Genga et al., 2012; Tomasi & Lupi, 2017; Li et al., 2012). Furthermore, the element with the second highest loading in the PC2 is Na, meaning that these two elements are highly correlated. Given the high loading of Cl and its correlation with Na, the second factor is associated with two sources of Cl: garbage burning and marine aerosols.

The third factor (PC3) explains 10% of the total variance with K having the highest loading. Potassium is commonly associated with biological matter (Zeb et al., 2018) as well as with biomass combustion (Amici et al., 2011; Ilacqua et al., 2007); therefore, a plausible source for PC3 would be the occurrence of wildfires. Further inspection of this factor shows that Mg and Na are two other elements appearing to have a contribution in PC3 (with loading < 0.7) having correlation with K. When potassium (in particulate matter) is associated with potassium, magnesium, sodium and/or iron, it could be related to soil dusts.

The fourth factor (PC4) explains 9% with sulphur having the highest loading, followed by Ca (however with a loading < 0.7) indicating the possible presence of calcium sulfate. The particles with sulphates in their composition are generated by its precursor SO_2_, which is emitted during the burning of fossil fuels and by biomass. SO_2_ can be absorbed onto the surface of mineral particles and form secondary aerosols (Li & Shao, 2009).

The remaining factors (PC5 and PC6) represent 17% of the variability, representing sources of Ti and Cu, respectively. These elements are without association, indicating that they are found as traces in the particles without a defined relationship. The identified source of titanium is soil dust, however, as it is present in little abundance, there is no preferential type of mineral matter, occurring in different forms from aluminosilicate clays rich in Fe and Ti or montmorillonite (Uddin, 2018). Copper could come from industrial processes and vehicle activity such as the abrasion of rubbers, lubricants and the corrosion of vehicle parts (Lough et al., 2005; Potter et al., 2021).

Figure S3 in supplemental material presents the dendrogram of the hierarchical cluster analysis (HCA). It shows that carbon is not associated with elements of mineral origin and that Si, O, Al and Fe are associated with each other; therefore, a group is identified and classified as belonging to the aluminosilicates or mineral matter of the soil. The elements Mg, K and Ti show a weak association to the group of aluminosilicates, so it is reasonable to think that they are present as traces in the mineral matter of the soil. The elements Na and Cl are associated in a group that is attributed to marine aerosols such as sea salt, being garbage burning another plausible source. Finally, an association of Ca and S is presented, possibly indicating the presence of particles with calcium sulphate. Considering that (resuspended) soil dust accounts for a major source of PM, results of SEM–EDX analysis agrees with local geology, that indicates microcline-quartz-muscovite gneiss which contains minerals of quartz, microcline and/or and muscovite.

To further develop on how the characterization of soil dust influences PM characterization, previous studies on the interaction of PM and other urban sediments (street/soil dust) and anthropogenic activities in Venezuela were revised. In the Metropolitan Region of Caracas, Suarez et al. (2004) using total reflection X-ray fluorescence, analysed dust samples recollected directly from the streets in several places of the city, including the Sartenejas Valley. Elemental content in environmental samples showed Fe, Sn and Pb had the highest concentrations. Automotive activity and soil dust were the proposed sources of iron. Handt et al. (2008) analyzed dust samples from different schools in Caracas. Industrial and vehicular emissions sources were associated with high concentrations of carbonate, Fe–Mn oxides and Ni. No recent studies have been conducted in the study area; however, the bibliography allows for the identification of vehicular activities as relevant in characterizing soil dust in the Sartenejas Valley. Some studies conducted in other parts of the country include Gamboa et al. (2020), Gamboa and Álvarez (2018) and Machado et al. (2008). Interaction of PM_2.5_ and other urban sediments is a topic that has been studied in Venezuela to little extent.

Morantes et al. (2021) showed a characterization and source appointment of PM samples restricted to the rainy season of 2018, in the Sartenejas Valley. Similar elemental compositions and sources were identified in both studies: Al-Si-related and Cl-related particles account for the majority of the variance of the PCA analysis. Ca-S and Ti-related particles were identified in both cases. However, in the present study, when accounting for a longer sampling period and by studying the elemental composition through a categorization by events, some differences arrived: the presence of K is related to two sources (forest fires and soil dust) and the presences of Cu-related particles are identified and associated with vehicular activities.

The PM physicochemical characterisation and source appointment (i.e. the PCA and HCA via elemental composition of particles obtained through SEM–EDS) is related to the logistic model results as both identify forest fires and high vehicle flow as predominant PM pollution events in the Sartenejas Valley.

### Proposal for early warning system in the surroundings of the Sartenejas Valley

The logistic model allowed us to identify that the variables with the greatest influence on the exceedances of PM_2.5_ are forest fires and vehicle flow. This finding is complemented by SEM–EDS/source appointment analysis by occurrence of events that showed that, when the threshold was exceeded, the predominant sources of aerosols were forest fires and the flow of motor vehicles which resuspend soil dust. It also identified the burning of garbage and the presence of biological dust as emission sources. Furthermore, the SEM–EDS/quantitative source appointment analysis showed that soil dust, garbage burning/marine aerosols and wildfires are three majority sources of PM. All of this is now taken into consideration to design an early warning system, regarding the increase in the concentration of PM in the air in the surroundings of the Sartenejas Valley and, in this way, control the adverse effects of this pollutant on health.

During the dry season, forest fires in the green areas of the Sartenejas Valley (~ 165 hectares) are frequent and could be of great magnitude, with potential for haze episodes (Foghin-Pillin, 2012, 2015). The Non-Governmental, universitary Organization Guardabosque along with the University Firefighters, have overseen the maintenance and recycling of organic waste from these green areas (the former), and the latter, of controlling and putting out the fires. These two organizations have very few financial and human resources, so it is necessary to dispose of these to plan to prevent the wildfires. The Food and Agriculture Organization (FAO) proposes a comprehensive wildland fire management handbook (Heikkilä et al., 2007) as a preventive measure for the construction and maintenance of firebreaks and the constant removal of dry organic matter. These measures have proven to be effective in reducing the losses of trees caused by fires, reducing their magnitude, and facilitating the access and initial work of the personnel who attack the fire fronts. Clearing shrubland (Lasanta et al., 2018), prescribed burnings (Silva et al., 2010), agricultural fields as fire breaks (Lloret et al., 2002) are also valid approaches towards wildfires mitigation. Finally, it is recommended to promote public environmental education as prevention measures to collaborate in the formation of citizens with the ability to understand the environment and respect it with a critical, autonomous attitude and willingness to change, for that, it is proposed to work together with the Network of Non-Governmental Environmental Organizations of Venezuela (in spanish, Red ARA) that execute programs from different perspectives (Directory of Environmental NGOs of Venezuela, 2012) and with universities, through community service programs that university students must attend before the end of the degree, in order to carry out environmental awareness campaigns on smoking, bonfires, logging, arson and fire lighting, to raise awareness of the danger posed by fire in forests and the damage it causes.

Vehicle emissions come mainly from resuspended soil dust, which are relevant for the air quality in urban environments (Amato et al., 2009; De la Paz et al., 2015; Piscitello, et al., 2021). Among the mitigation measures to reduce dust emissions by resuspension on highways are i) Wet sweeping of the streets, with water or dust suppressing agents, using mechanical or vacuum sweepers; ii) Street washing with cisterns (automatic or manual) at times adapted to the local vehicular behaviour (in Sartenejas Valley, 04h00-05h00) (Amato et al., 2014; Norman & Johansson, 2006; Querol et al., 2017). Preventive measures related to urban planning policies (Piscitello et al., 2021) must also be taken, such as iii) Reinstate the vehicle traffic restriction measures from Monday to Friday from 6h30 to 9h30, through the “*Plan Pico y Placa*” (driving restriction policy aimed to mitigate traffic congestion), operative until 2007, when it was suspended by Sentence No. 01267 of the Supreme Court of Justice, by a divergence between the local and central government; iv) Higher frequency in the maintenance of potholes resulting from heavy rains and the continuous passage of trucks on motorways with greater traffic; v) Measures for paving dirt roads with low traffic, characteristic of the poorest neighbourhoods.

Open waste burning is spurred by a lack of systematic waste collection that results from an incompetent waste management system; therefore, communities deliberately burn garbage as an alternative for final disposal. It is a problem globally, specifically in developing countries, including Latinamerica (Ferronato & Torretta, 2019; Cogut, 2016; Sáez & Urdaneta, 2014). In the Metropolitan Region of Caracas, according to the EPE II (2018), the generation of garbage is 1.1 kg-inhab-1.day-1. The processes of separation at the origin, characterization, storage and recycling are practically not present (only 2% of the total garbage produced is recycled, INE, 2010), compared to the average for the Latin American and Caribbean region, which is about 12%. In addition, the coverage of garbage collection at the national level is 75%. The constant failures in the garbage collection service force citizens to dispose of garbage on public roads (21.9%), open waste burning (15.5%), private collection to undisclosed locations (14.4%) or bury it (1.5%) (García, 2020). Contaminants released during garbage burning/waste burning can include HCl (Cogut, 2016), heavy metals, petroleum hydrocarbons, semi-volatile organic compounds (SVOC), polychlorinated biphenyls (PCBs), dioxins and furans (Pérez et al., 2013). Reducing the amount of garbage burning HCl-related emissions can be made by reducing/eliminating the usage of plastics (for instance in grocery packaging) which is already promoted as an environmentally friendly alternative to the matter: this measure should be part of the environmental education program. To reduce emissions related to open waste burning, special attention should be paid to improving integrated solid waste management systems that include landfill management and prevention through education to communities on the hazards of open waste burning (Coffey & Coad, 2010) as formulated within the current national law (República Bolivariana de Venezuela, 2010).

Regarding bioaerosols, it is almost impossible to diminish the presence of biogenic particles in the Valley, due to the 165 hectares of green areas that make up it. Therefore, there is going to occur a constant release of pollen, which also, being a humid and cool area, it is favoured the proliferation of airborne mites and fungi in the season that coincides with the flowering and pollen release (Perdomo de Ponce, 2009), exacerbating allergies in the respiratory and ocular mucosa’s, generating nasal obstruction, sneezing, wheezing, asthma, and allergic rhinitis (Parmes et al., 2020; Thibaudon and Besancenot, 2019; Dudek et al., 2018). These symptoms decrease the quality of life of people prone to allergic reactions, particularly in those that have not developed an immunologic tolerance to ambient allergens (Pawankar et al., 2011; Ruokolainen, 2017). Because pollen is of natural origin, it only remains to take preventive measures to reduce its effects, such as maintaining windows closed during high pollen episodes, wearing glasses in the street as a protective barrier in early and late hours of the day, and cleaning the houses with damp rags to avoid dust dispersion.

Finally, once the fires have occurred, or after verifying an increase in PM concentration due to one of the other causes already mentioned, lineaments must be established for individual and population level protection from particulate exposure. It could be suggested that elderly and vulnerable populations not do outdoor activities; restrict school breaks to closed areas; those suffering from respiratory diseases must wear face masks outside and avoid exercising outdoors. The application of an EWS at the study area must be accompanied by an air quality monitoring system with information to the population in real-time.

The current Venezuelan reality presents a socioeconomic crisis (OEA, 2018) that limits the design of a preventive EWS based on geographic information systems, simulators and big data. EWSs are usually entangled with national-or-local air quality monitoring networks. In Venezuela, the National Institute of Statistics (INE, in its acronym in Spanish) by 2021 only showed information on air quality for the metropolitan region of Caracas updated till the year 2010 (INE, 2010). The information published comes from an TSP-only air quality network made up of six monitoring stations distributed in the city of Caracas: Los Ruices, Bello Campo, El Cementerio, La Yaguara, La Trinidad and El Silencio (Guajardo, et al., 2010). This reflects the lack of current information on air quality nationwide. Morantes et al. (2016) reported that by 2014 there was no readily accessible public information on air quality in the country. Furthermore, the air quality regulations in Venezuela have not been updated since 1996, which only regulates TSP. Moreover, not only the lack of information seems to be a reality, as the lack of formal environmental education has been established to make it difficult to face main environmental problems in the country (INE, 2010) with an evident lack of current formal institutional reports on the matter. The lack of readily official information on air quality makes it that much more important to address independent studies on the matter, that usually come from academia.

This reality forces us to narrow the proposal to measures that help reduce the consequences. To date, the levels of PM_2.5_ pollution in the study area are low; however, an EWS must be put in place to prevent a progressive increase in pollution episodes, particularly in the dry season when consecutive wildfires occur, and the recommended WHO-2021 PM_2.5_-24 h threshold is exceeded with more frequency. The given recommendations were chosen considering the feasibility and logistics of implementing actions in these areas given the contextual reality.

## Strengths and limitations

The main strength of this research is extending on existing studies of outdoor PM_2.5_ sampling and characterization in Venezuela. Bibliography shows there is a scarcity of studies of PM in cities of Venezuela, some of which date prior to 2010. This comes particularly important when accounting for the lack of public, up-to-date information on air quality in the country, therefore, academic studies come to fill the need for information regarding PM air pollution and the events that influence it.

The main limitation is that for reasons of equipment availability, the study only relies on one sampling station of PM_2.5_. This will almost completely ignore atmospheric PM2.5 sources in areas remote to the location chosen for the study. Although this limitation is considered, the authors have stated that the location of the sampling station was chosen to be representative of the area and the results indicate that different sources of PM were accounted for. Another limitation is the number of samples taken. Although 41 samples would seem likely to be under representative for an air quality study, the authors make the argument that the sampling times and period accounts for one year of sampling, which includes climatological and seasonal variations, weekday and weekend variations, being in line with the recommendations for air quality sampling established in the national regulation on air quality (Morantes et al., 2019). Regarding pollution episodes (i.e. concentrations exceeding the current WHO-2021 recommendations and ~ 7 DALYs per 1,000 people) several episodes were registered during the sampling period, particularly responding to the wild-fire season. Supplemental information (Figure S2) shows that for a previous year of sampling, pollution episodes were also measured during the season. When considering that the threshold for the logistic model was set as half the WHO-2005 guideline for PM_2.5_-24 h, being just 2.5 absolute points below current WHO-2021 guidelines, goes to show that the magnitude of the PM_2.5_exc_12.5_ value was reasonable.

## Conclusions

The logistic model’s findings and the SEM–EDS analyses support evidence that, in the Sartenejas Valley, an area with low levels of PM_2.5_, forest fires and high vehicle flow contributed significantly to increased concentrations of PM_2.5_. This conclusion is relevant for risk assessments when considering the relationship between increased PM_2.5_ and health burden. Therefore, forest fire and vehicular traffic events should be used as inputs when designing early warning systems.

Although particle concentrations in the area are below the current WHO-2021 PM_2.5_-24 h guideline value, the bi-variate analysis, the regression results and the particle characterization allowed us to identify the variables that significantly influence PM pollution events in the Sartenejas Valley surrounding.

Elemental composition analysis of PM (via SEM–EDS) identified biomass burning from forest fires and resuspended dust from vehicular traffic as the two dominant sources of pollution in days with the highest PM_2.5_ concentrations levels. During forest fire events, filters were saturated, and the elemental composition indicated high carbon content with traces of potassium. The morphological analysis allowed us to identify small and spherical particles that are characteristic of combustion processes, complex agglomerates, and atmospheric mixing processes. In days with lowest levels of PM_2.5_, the morphology of the particles showed ordered shapes indicating their biological origin, and the elemental composition indicated minerals from the soil. Source appointment via PCA shows that soil dust, garbage burning/marine aerosols and wildfires are three sources of PM explaining ~ 50% of the variance.

The proposed early warning system for PM pollution in the surroundings of the Sartenejas Valley should include preventive measures related to fire breaks to avoid the proliferation of wildfires. These measures require funding from the local and central government. Likewise, it is proposed to promote environmental education as a tool for the prevention of forest fires. Regarding PM_2.5_ from resuspended dust, a series of mitigation measures are proposed that only local governments can implement related to road maintenance. Even though the burning of garbage is not part of the exceedance model, the elemental analysis revealed the presence of Cl, possibly from the burning of garbage. To avoid the habit of burning garbage in the open air, it is essential to modify the city's garbage collection system, which, not being able to properly manage solid waste, forces citizens to reinvent what to do with them. Finally, as the Sartenejas Valley is an area with many green areas, the only way to avoid allergies caused by outcrop and pollen is by taking the preventive measures indicated in the proposal. Actions to cope with an increase in PM_2.5_ concentration due to any of the causes already mentioned, are included within the recommendations. The overall success of the EWS proposal requires the leading role of the local and central government as well as the empowerment of citizens in order to carry it out.

## Supplementary Information

Below is the link to the electronic supplementary material.

Supplementary file1 (DOCX 779 KB)

## Data Availability

The datasets used and/or analysed during the current study are available from the corresponding author on reasonable request.

## Footnotes

1 WHO: World Health Organization; USEPA: United States Environmental Protection Agency.
