# Supplementary material for: PM2.5 exceedances and source appointment as inputs for an early warning system
Source: Environ Geochem Health. 2022 Feb 22;44(12):4569–93. doi: 10.1007/s10653-021-01189-2 (PMC9675665; doi:10.1007/s10653-021-01189-2)

**Concentrations, source appointment and exceedances of ambient PM2.5 as inputs for an early warning system**

Supplemental Material.

Figure S1. Geological Map of the Caracas Metropolitan Region


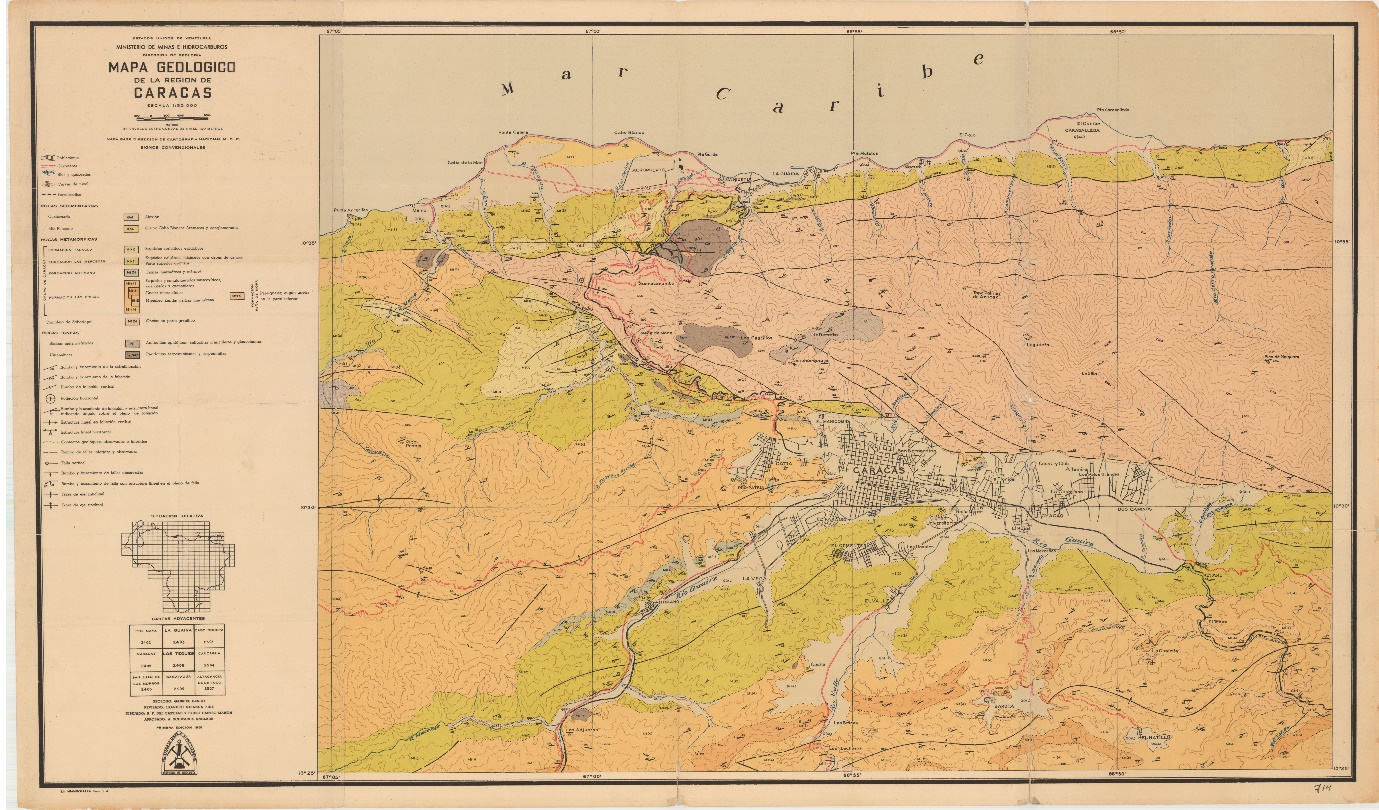


DENGO, G. (1951). Mapa geológico de la región de Caracas, escala 1:50.000. Boletín Geológico, Caracas, 1 (1): 39-115.

Figure S2. Sartenejas Valley PM2.5-24h concentrations and events (2014 to 2015)


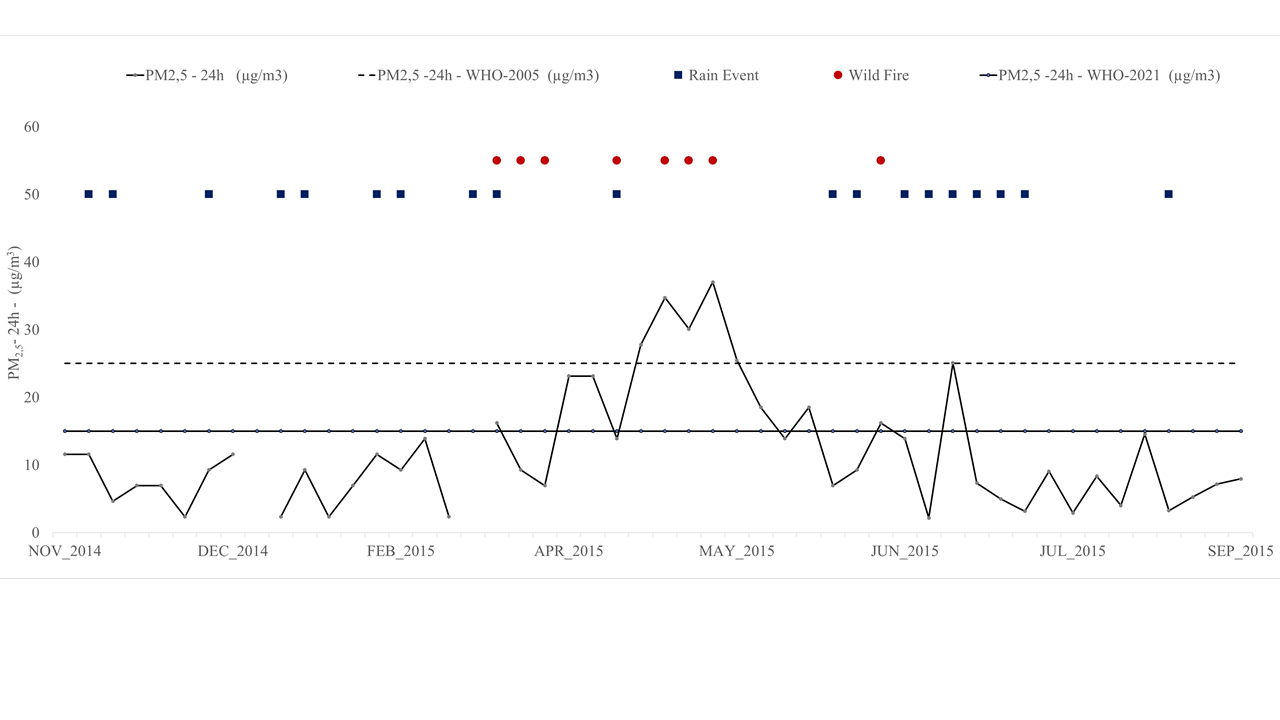


Figure S3. Dendrogram of hierarchical clusters of the elemental composition of PM2.5 particles


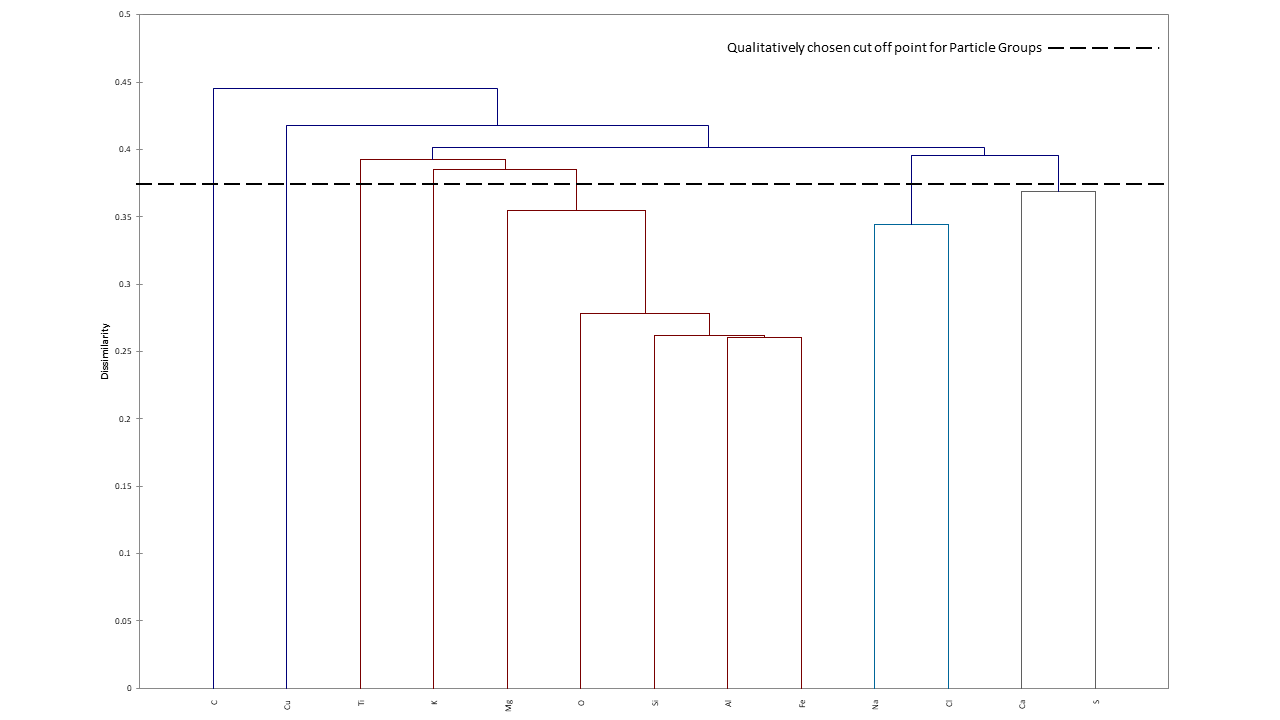

Supplement: Supplementary file 1 — Supplementary file1 (DOCX 779 KB) [file 10653_2021_1189_MOESM1_ESM.docx]
